# A qualitative analysis of feelings and experiences associated with perinatal distress during the COVID-19 pandemic

**DOI:** 10.1186/s12884-022-04876-9

**Published:** 2022-07-18

**Authors:** Katie Jones, Virginia Harrison, Michelle L. Moulds, Lisa Lazard

**Affiliations:** 1 School of Psychology and Counselling, The Open University, Milton Keynes, UK; 2 School of Psychology, UNSW Sydney, Sydney, Australia

**Keywords:** Perinatal distress, Perinatal anxiety, Perinatal depression, Antenatal, Postnatal, Mental health, COVID-19

## Abstract

**Background:**

Rates of perinatal mental health difficulties (experienced during pregnancy and the 12-months postpartum) increased worldwide during the COVID-19 pandemic. In the UK, anxiety and depression were estimated to affect more than half of perinatal women during the first national lockdown. However, little is known about women’s qualitative experiences of distress. This study aimed to extend published quantitative findings resulting from the same data set (Harrison et al., Women Birth xxxx, 2021;  Harrison et al., J Reprod Infant Psychol 1–16, 2021) to qualitatively explore: 1) the feelings and symptoms associated with maternal perinatal distress during the COVID-19 pandemic; and 2) the associated sources of distress.

**Methods:**

As part of an online survey during May 2020, 424 perinatal women responded to an open-ended question regarding a recent experience of distress. Qualitative data were analysed using an initial content analysis, followed by an inductive thematic analysis adopting a realist approach. Data were explored in the context of self-reported perinatal anxiety and depression symptoms.

**Results:**

Initial content analysis of the data identified twelve distinct categories depicting participants’ feelings and symptoms associated with psychological distress. Despite the high rates of probable depression in the sample, women’s descriptions were more indicative of anxiety and general distress, than of symptoms traditionally related to depression. In terms of the associated psychosocial stressors, a thematic analysis identified five themes: *Family wellbeing; Lack of support; Mothering challenges; Loss of control due to COVID-19;* and *Work and finances*. Unsurprisingly given the context, isolation was a common challenge. Additionally, psychological conflict between maternal expectations and the reality of pregnancy and motherhood, loss of autonomy and control, and fears surrounding family health, safety, and wellbeing underlay many of the themes.

**Conclusions:**

This study presents an array of feelings and symptoms expressed by perinatal mothers which may be useful to consider in relation to perinatal wellbeing. Furthermore, our data highlights several common sources of distress, including multiple COVID-19 specific factors. However, many were related to more general perinatal/maternal experiences. Our findings also point to considerations that may be useful in alleviating distress in pregnancy and early motherhood, including social support, realistic perinatal/maternal expectations, and support for those with perceived perinatal trauma.

## Background

Pregnancy and childbirth represent major transitions in a woman’s life, increasing vulnerability to emotional distress and perinatal mental health (PMH) difficulties [1]. Prior to 2020, approximately 25% of mothers experienced a psychological disorder such as anxiety or depression during pregnancy and/or the year following childbirth [2], however rates increased during the COVID-19 pandemic [3, 4]. In the UK, the prevalence of symptoms of clinically significant perinatal depression was reported to have reached 43–49%, whilst rates of anxiety symptoms were 49–61% [5–7], far higher than the global pooled prevalence during the pandemic of 25.6% for depression and 30.5% for anxiety symptoms [4]. There is considerable comorbidity between symptoms of perinatal anxiety and depression [8], both of which are reported to negatively affect mother and child wellbeing with a combined cost to the UK of approximately £6.6 billion per year (reported prior to the COVID-19 pandemic [9]). Researchers have predominantly used quantitative methods to investigate the prevalence and risk factors associated with clinically relevant symptoms. Accordingly, the qualitative experiences of anxiety, depression, and more general psychological distress during the perinatal period are less understood.

A disproportionate amount of PMH information, support, and screening tools focus solely on postnatal depression [10], and perinatal women may struggle to identify other psychological symptoms [11, 12]. Moreover, there is evidence that a fear of stigma is related to limited disclosure of symptoms [11, 13, 14]. An increased understanding of perinatal women’s experience of psychological distress may help to guide information and criteria used to identify women in need of support. Indeed, a systematic review and meta-synthesis of studies in the UK reported barriers to PMH support existing on four levels: individual, organisational, sociocultural, and structural [15]. For example, a lack of understanding of signs and symptoms of PMH difficulties was reported amongst healthcare professionals, perinatal women, and family members, and symptoms were often dismissed as normal perinatal experiences. When considering how support is provided, it may be more fruitful to focus on more relatable and transdiagnostic constructs that are independent of diagnosis, such as ‘distress’ [12, 16, 17]. Regardless of the terminology adopted, negative effects of PMH difficulties on mother-child interactions (e.g., [18–20]) and long-term adverse consequences for the child (e.g., [21–23]) underpin the need for greater understanding of the qualitative nature of perinatal distress and sources which may trigger such experiences.

An accumulation of factors is theorised to increase susceptibility to PMH difficulties [24]. However, less is known about the specific events and experiences which may trigger psychological distress. The COVID-19 pandemic introduced an array of stressors likely to increase the risk of PMH difficulties [25–27] and evidence is gradually emerging to support these early predictions. For example, fear of contracting the virus and its consequences presented a particular worry for pregnant women, who were initially considered more vulnerable to COVID-19 than the general population [28–31]. Furthermore, women faced uncertainty around perinatal care [7, 30, 32, 33], Health Visitors were redeployed in some parts of the UK, and many families reported not experiencing the perinatal care that they had expected [33, 34]. Lockdown instigated to limit transmission of the virus resulted in extended periods of physical and social isolation, preventing access to many forms of support, and leaving co-parents excluded from attending antenatal appointments or visiting mothers during postpartum hospital admissions [26, 30, 32, 33].

A perceived lack of social support has been repeatedly associated with increased risk of perinatal psychological disorders (e.g., [35, 36]) which may, in part, explain increased rates of psychological symptoms during the pandemic [5, 6]. Another explanation may be rooted in the mismatch between maternal expectations and reality. Unmet maternal expectations have previously been associated with increased perinatal psychological distress outside of the context of COVID-19 [11, 16]. Unattainable idealised expectations of childbirth and social norms in early motherhood [11], and unrealistic beliefs about postnatal care often reported by first-time mothers [37] may underlie this association. Furthermore, a range of distressing experiences common to the transition to motherhood, including difficulties coping with increased demands and challenges, changes to relationships and the social context, and adapting to becoming a mother, may also contribute to maternal distress [38].

While the prevalence of PMH difficulties has increased during COVID-19 [3, 4, 7], further research is required to understand the specific nature of psychological distress experienced by perinatal women in the UK. Although a small number of qualitative studies of perinatal mental wellbeing have been published globally during the pandemic, it is difficult to draw comparisons across countries given that government responses to COVID-19 have varied greatly. To our knowledge, the qualitative research conducted in the UK has, to date, focused on very specific aspects of perinatal experiences (e.g., [33, 39]). The current study qualitatively explores descriptions of personal experiences of perinatal distress during the first UK lockdown. As well as adding to the small volume of qualitative research describing symptoms of perinatal distress (e.g., [12]), identifying the feelings and symptoms associated with distress during the pandemic may be valuable to the development of interventions aimed at reducing their long-term impacts. For example, previous literature [11, 12, 15] has noted that a greater understanding of context-specific symptoms is necessary to appropriately target support. Furthermore, analysing individual descriptions of commonly occurring sources of distress has scope to inform psychological interventions for women who report psychological distress in the context of the pandemic.

Accordingly, in the current study we aimed to 1) qualitatively explore the feelings and symptoms perinatal women report being associated with psychological distress in the context of COVID-19; and 2) reveal the experiences that perinatal women associate with increased feelings of distress during the pandemic.

## Methods

### Design

We conducted an initial content analysis, followed by a thematic analysis of data from an open-ended survey question embedded within a large online mixed-methods survey in the UK during the COVID-19 pandemic. Qualitative surveys are useful for investigating under-explored phenomena due to their ability to efficiently capture meaningful data relating to diverse individual experiences from large samples [40]. The anonymous nature of online surveys may be particularly beneficial when addressing sensitive topics, potentially reducing social desirability biases and fear of stigma. We therefore reasoned that a qualitative survey may increase disclosure of symptoms and encourage previously unexpressed details of distressing experiences to be shared. Given this study was conducted during the pandemic, online methods were deemed an appropriate and acceptable medium. They were also necessary, as the lockdown restrictions in place at the time of data collection precluded in-person contact.

### Participants and procedure

A convenience sample of 456 perinatal women was recruited through social media, forums, and companies, and via the participant recruitment service Prolific (www.prolific.co). Participants responded to an advert for a larger piece of research stating that we were looking for pregnant women and those in the first year after childbirth to take part in a study investigating “maternal wellbeing, social support and technology use” during the Coronavirus pandemic (previous quantitative findings are reported elsewhere ([5, 6]). Inclusion criteria specified women needed to be: 1) pregnant or within 12 months postpartum; 2) aged 18-years and over; 3) living in the UK; and 4) fluent in English. All participants who completed the questionnaire were entered into a prize draw for one of three £20 Amazon vouchers.

Participants anonymously completed the online questionnaire in May 2020. A subset of 424 women (93%) responded to the following open-ended question, providing data for this analysis:

*We want to better understand how people experience mental health issues in the perinatal period, as this may help us to learn how to better support women at this time. To do this, we want you to think about the last time you felt especially distressed or upset. Briefly describe this situation in terms of what happened (i.e., what was the reason for your distress or upset) and what you did.*

Participants also reported demographic data and completed a range of standardised self-report measures. We include this data as a means by which to characterise the sample as per Newby et al., [41]. Results are displayed in Table 1. Quantitative analyses of the remaining data obtained from the survey to address other research questions (i.e., about the role of repetitive negative thinking in the perinatal period) are published elsewhere [5, 6].

**Table 1:** Demographic information

	Pregnant women (*N* = 190)	Postnatal women (*N* = 234)
	*N* (%)	*N* (%)
**Age**		
18–24	12 (6.3)	10 (4.3)
25–34	121 (63.7)	150 (64.1)
35–44	57 (30.0)	74 (31.6)
**Education**		
GCSEs or equivalent	12 (6.3)	15 (6.4)
A Levels or equivalent	37 (19.5)	43 (18.4)
Undergraduate degree	70 (36.8)	92 (39.3)
Postgraduate degree	65 (34.2)	79 (33.8)
Other	5 (2.6)	5 (2.1)
**Relationship**		
Married or cohabiting	182 (95.8)	227 (97.0)
Single	4 (2.1)	4 (1.7)
Non-cohabiting partner	3 (1.6)	3 (1.3)
**Living arrangements**		
Living alone		1 (0.4)
Living alone with child/ren	4 (2.1)	6 (2.6)
Live with partner and child/ren	103 (54.2)	224 (95.7)
Live with partner and no children	77 (40.5)	3 (1.3)
Live with parents and/or siblings	4 (2.1)	
Live with partner and extended family	2 (1.1)	
**Employment**		
Full-time employment	79 (41.6)	24 (10.3)
Part-time employment	35 (18.4)	21 (9.0)
Self-employed	11 (5.8)	13 (5.6)
Studying	4 (2.1)	1 (0.4)
On maternity or sick leave	10 (5.3)	151 (64.5)
Furlough	22 (11.6)	2 (0.9)
Not in paid employment	20 (10.5)	22 (9.4)
**Ethnicity**		
Any White background	171 (90.0)	221 (94.4)
Mixed, or multiple ethic groups	8 (4.2)	4 (1.7)
Asian, or Asian British	6 (3.2)	6 (2.6)
Black African, Black Caribbean, or Black British	4 (2.2)	2 (0.8)
Any other ethnic group	1 (0.5)	1 (0.4)
**Recruitment**		
Social media	138 (72.6)	181 (77.4)
Prolific	52 (27.4)	53 (22.6)
**Trimester**		
1st	65 (34.2)	
2nd	64 (33.7)	
3rd	61 (32.1)	
**Months since childbirth**		Mean (SD) 6.32 (3.38)

### Measures

The *Edinburgh Postnatal Depression Scale (EPDS)* is a 10-item self-report measure of perinatal depressive symptoms [42]. Respondents rate the extent to which each item applied to them over the past week using a 4-point Likert-type scale which provides different answers for each question; for example: ‘*I have felt sad or miserable, (1) Yes, most of the time; (2) Yes, quite often; (3) Not very often; or (4) No, not at all’*. Scores range from 0 to 30, with scores ≥13 considered to reflect probable depression in the context of research. Cronbach’s α = 0.87, indicating high reliability [42].

The *Perinatal Anxiety Screening Scale (PASS)* is a 31-item measure of perinatal anxiety symptoms [43]. Respondents report how often they have experienced each of the statement (such as ‘*feeling overwhelmed’)* using a 4-point Likert scale with the options of (1) not at all; (2) sometimes; (3) often; (4) almost always. Scores range from 0 to 93 with scores ≥26 suggesting probable anxiety. Furthermore, scores between 21 and 41 indicate mild-moderate anxiety, and scores between 42 and 93 suggest severe symptoms [44]. It possesses excellent construct validity and reliability (Cronbach’s α = 0.96 [43];).

### Data analysis

First, a content analysis was conducted following Elo and Kyngas’ ([45]) methodology as a means of contextualising the data, highlighting any prominent patterns in the language used relating to participants’ feelings. Data from the open-ended survey question were then analysed using inductive thematic analysis to explore the experiences participants associated with distress during the pandemic. We adopted a realist approach to the thematic analysis of the data following Braun and Clarke’s [46] guidelines. This approach makes the ontological and epistemological assumptions that while reality exists (ontological realism), it can only be accessed indirectly as it is processed by us and understood within the confines of our social, psychological, cultural, historical, and linguistic context. The aim of the thematic analysis was to identify experiential themes in the data that captured participants’ experience of perinatal distress during the pandemic (such as their feelings, concerns, and beliefs), and analysis was carried out at the semantic level. Experiential themes were generated inductively by reading through each participant response and annotating them with simple semantic codes representing their experiences based on key words and phrases within the data to maintain focus on participants’ own expressions. As more responses were read (and in subsequent readings of the data) code generation also became deductive, as responses were checked against previously coded responses. These semantic codes were discussed between authors to confirm they were appropriately represented in the data. Relevant patterns were identified within the data and codes were collated to form meaningful experiential themes, and thematic maps were developed to guide the creation of themes and subthemes. These were continuously refined, taking care to give each extract equal priority and avoid a limited number of vivid examples influencing the analysis. Quotations were reviewed in the context of the entire participant response to ensure they retained their original meaning. The analysis was subject to qualitative methodological criteria for rigour by the research team including the extent to which the analysis was plausible and transparent.

Following complete thematic analysis, the prevalence of each theme and subtheme was explored within the antenatal and postnatal sub-samples to investigate differences between the two groups.

## Results

### Self-report measures

The self-report measures described above provide important context for the qualitative analysis of the open-ended survey question that formed part of the overall survey. Specifically, the responses to these measures (Table 2) indicated that almost two-thirds of respondents scored in the clinical range for anxiety or depression (EPDS ≥13 and/or PASS ≥26).

**Table 2:** Anxiety (PASS) and Depression (EPDS) symptom scores

	Pregnant women (*N* = 190)	Postnatal women (*N* = 234)
	*N* (%)	*N* (%)
Clinically concerning depression symptoms (EPDS ≥13)	86 (45.3)	116 (49.6)
Clinically concerning anxiety symptoms (PASS ≥26)	93 (48.9)	114 (48.7)
Clinically concerning anxiety and/or depression symptoms (EPDS ≥13 and/or PASS ≥26)	114 (60.0)	148 (63.2)
Mild to moderate perinatal anxiety symptoms (PASS 21–41)	87 (45.8)	100 (42.7)
Severe perinatal anxiety symptoms (PASS 42–93)	36 (18.9)	46 (19.7)

### Content analysis: feelings and symptoms associated with maternal distress during the perinatal period

Of 424 women who completed the open-ended survey question, 73% (*N* = 310; 45% prenatal, 55% postnatal) described their feelings and symptoms associated with distress. Content analysis was used as a method of initially organising the data and to highlight prominent patterns in the language used to describe experiences and associated feelings that could otherwise be missed (e.g. [47]). In this study, content analysis generated twelve categories of feelings and symptoms described by respondents, detailed in Table 3 and listed in order of prevalence within the combined perinatal dataset. Just over one-third of the respondents included in this analysis contributed data to multiple categories. This suggests that conceptual distinctions between particular subjective feelings become interwoven in specific contexts of experience. We report the prevalence of each category amongst all respondents, and for the prenatal and postnatal sub-samples separately to highlight any comparisons between these groups.

**Table 3:** Feelings and symptoms associated with distress during the perinatal period

Category	Example	Percentage (n) of prenatal women ***N*** = 141	Percentage (n) of postnatal women ***N*** = 169	Percentage (n) of perinatal women N = 310
Upset and tearful^a^	My partner couldn’t come to my ultrasound appointment. I cried and got very upset (A171)	41 (58)	56 (77)	44 (135)
Worry and overthinking	I was worried he might have caught Covid-19. I wasn’t able to contact the GP and had to take my baby to A&E. It turned out he was ‘just’ teething. I was very distressed and upset, because I was thinking about the worst even when it was nothing serious (P40)	28 (39)	26 (44)	27 (83)
Fearful and scared	Made me feel in danger for my life and babies, very distressed and struggled to carry on working (A101)	16 (22)	10 (17)	13 (39)
Guilt, failure, self-blame, and inadequacy	I was triggered by all conversations I have with anyone. I feel vulnerable about sharing my feelings and cry and it makes me feel guilty and ashamed. I want to turn back time and relive it better because I end up regretting having the energy or not saying the right things. I get distressed and upset over everything. I can’t reach expectations or other people’s and just want to be invisible (P249).	8 (11)	14 (24)	11 (35)
Anxiety and nervousness	I hadn’t felt the baby move for a while, all the anxiety of fertility treatments and miscarriages came back to me (A106))	11 (16)	10 (17)	11 (33)
Stressed	My baby had a nosebleed and I was stressed out because I had to go to the local hospital (P2)	11 (15)	8 (14)	9 (29)
Frustrated, agitated, and disappointed	Doing housework and feeling unsupported by my husband who just wanted to spend the day doing nothing (sleeping, watching tv, relaxing). We had a brief argument where I voiced my frustrations (A41)	9 (13)	7 (12)	8 (25)
Panic	I put her down and have a meltdown, it’s like I’m screaming on the inside, like I’m rushing, panicked. (P116)	7 (10)	7 (11)	7 (21)
Overwhelmed and unable to cope	I felt extremely overwhelmed and just wanted to hide away and ignore her and the world P194	5 (7)	7 (12)	6 (19)
Sad and low	I woke up just feeling generally down. My husband was the same so neither of us really talked and when we did it was snippy. I cried, I took deep breaths, walked around the house aimlessly, tried to sleep it off. (A77)	8 (11)	4 (6)	5 (17)
Angry, irritated and on edge	I got so angry I was shaking and wandering round the house doing loads of chores to distract me (P218)	3 (4)	6 (10)	5 (14)
Nightmares and intrusive thoughts	Repetitive nightmares of my baby being taken away from me and i never got to see her (A182)	5 (7)	3 (5)	4 (12)

^a^*Upset and tearful* may be over-represented due to the wording of the survey question

Feeling upset and tearful was the most frequently expressed symptom associated with distressing experiences, and was repeatedly reported alongside a constellation of other emotions (Table 3). While it may seem plausible that crying was related to feeling low (or depressed feelings), this was not borne out in the responses. Instead, combinations of symptoms were common, with women often reporting crying when experiencing other emotions beside sadness, such as fear, anger, irritation, and frustration.

Interestingly, despite responses on the EPDS suggesting almost half of the participants in this sub-sample were likely experiencing depression (EPDS ≥13), feelings commonly associated with depression such as feeling sad, low, or withdrawn were relatively uncommon in comparison to feelings and symptoms more readily associated with anxiety (such as nervousness, worry, overthinking and fear). Indeed, worry and overthinking was the second-most prevalent category reported. The subject of these worries predominantly focused on present or future concerns rather than overthinking about past experiences (i.e., rumination), which was not surprising given the uncertainty about the future that surveyed women were facing, both in terms of the transition to motherhood and the COVID-19 pandemic. However, whether worry is necessarily indicative of anxiety in these participants is difficult to ascertain, as repetitive negative thinking is evident across an array of emotional disorders [48] and has been repeatedly linked to depression and other mental health issues. Therefore, it may be better conceptualised as a transdiagnostic indicator of psychological distress. Regardless, anxiety and nervousness were also described in relation to experiences of distress. In some cases, such feelings were extreme, described in terms of ‘panic’ and feeling ‘terrified’ or ‘petrified’, descriptors which highlight the intensity of these women’s experiences. For a small but concerning number of people, worry or overthinking was related to nightmares and intrusive thoughts, most often related to fears for the baby (Table 3).

Another feeling reported to be associated with distress was guilt, which was most apparent amongst postnatal respondents and commonly associated with feelings of *‘not being a good mum’* or *‘not doing enough’*. Again, these feelings often arose alongside other symptoms, such as frustration tearfulness and panic.

Content analysis of the data provided an initial frame which informed the thematic analysis of the data described below.

### Thematic analysis; salient sources of perinatal distress during the COVID-19 pandemic

Of the 424 participants who responded to the open-ended survey question, 89% (*N* = 377; 43% prenatal, 57% postnatal) attributed their feelings of distress to specific experiences. Thematic analysis yielded five themes and seventeen subthemes, detailed in Table 4 in order of prevalence within the combined perinatal dataset.

**Table 4:** Themes and subthemes describing salient sources of maternal perinatal distress reported during the COVID-19 pandemic

Theme	Subtheme	Example	Percentage (n) of prenatal women ***N*** = 163	Percentage (n) of postnatal women ***N*** = 214	Percentage (n) of perinatal women N = 377
**Family wellbeing**			**42 (69)**	**43 (91)**	**42 (160)**
	Concerns for the family’s health and safety during the pandemic	I frequently worry about either my baby catching covid and being ill or myself catching it and dying. The thought of leaving her to grow up without a mother stops me sleeping. (P41)	16 (26)	17 (36)	16 (62)
	Fear for the infant’s health, safety, and wellbeing	The fear of SIDS still scares me even 10 months down the line and despite knowing I have done everything to prevent it. I overthink what might happen and why and check on the baby regularly to ease my worry. This can include images of him being dead. (P18)	1 (1)	19 (41)	11 (42)
	Obstetric complications	Following a really painful egg retrieval as part of IVF- can’t get over it or let it go even though it was successful as it should not have been painful. (…) I am now frightened about birth where I was unconcerned/no more concerned than anyone else. I often cannot sleep reliving this experience and fear this may happen after birth and I will end up with postnatal depression or that something will go wrong in the pregnancy as it did in the egg retrieval. (A206)	10 (17)	4 (8)	7 (25)
	Fears for the pregnancy and birth	I’ve been significantly on edge all day, panicking and on the edge of tears because I am worried about the baby’s movements. I’ve been using an app to track them that’s got 200 individual movements registered on it for today alone but when she’s not moving I’m worried and when she’s moving I’m worried it’s not strong enough. It’s really hard to control the anxiety about her (A202)	13 (21)	< 1 (1)	6 (22)
	The unknown long-term implications of social isolation during the pandemic	Even though my child is only a year old all the recent changes due to the pandemic have made me worry that she is perhaps not getting enough socialization and that that could impact him further down the line. (P32)	2 (4)	2 (5)	2 (9)
**Lack of support**			**38 (62)**	**31 (66)**	**34 (128)**
	Isolation and a lack of social support	When baby is crying for long periods of time (teething or over tired) I … desperately need some outside support from family. I often get myself very worked up that my family are missing out on my son’s life (P163)	13 (21)	18 (38)	16 (59)
	Unsupportive relationships	Partner disrupted baby’s daytime routine at the weekend. Naps were then out of sync and he slept poorly that night meaning I was up several times as partner doesn’t help overnight. Felt frustrated that partner tries to undo all the hard work I put in and doesn’t recognise how disruptive it is. We landed up in a row and it dragged up old negative feelings about old problems with our relationship. (P162)	10 (17)	11 (23)	11 (40)
	Restrictions in perinatal care	At the first scan for our pregnancy, my husband was not allowed to even come in the hospital. Whilst I completely understood the rules, I am very anxious and nervous in hospitals at the best of times and would have loved his support. I was also incredibly sad that he missed such an important moment and couldn’t be there. I found being at the hospital daunting and because it was all unknown, I was very anxious and upset. (A17)	15 (24)	2 (5)	8 (29)
**Mothering challenges**			**13 (22)**	**35 (74)**	**25 (96)**
	Infant crying and sleep deprivation	The baby was crying and I couldn’t calm her, normally I’m hands on with calming her down but this 1 time I couldn’t and kept thinking something was wrong (P51)	0	16 (34)	9 (34)
	Difficulty achieving personal mothering expectations	Too many tasks/chores to do, whilst looking after baby and get upset at not keeping on top of things, plus constant stressing that I’m not doing enough with my baby. (P168)	7 (12)	10 (21)	9 (33)
	Competing demands on time	Felt really overwhelmed trying to home school my reception aged son and look after my premature newborn baby (P83)	6 (10)	9 (19)	8 (29)
**Loss of control due to COVID-19**			**14 (23)**	**7 (16)**	**10 (39)**
	Loss of normality	When I found out I was pregnant (even though it was planned) we had been trying for a long time. It seemed like the worst time for it to happen with Covid-19 and the restrictions on daily life. (A145)	4 (7)	5 (11)	5 (18)
	Feeling trapped	I then couldn’t stop crying and was so frustrated because we couldn’t go anywhere or do anything. (P219)	2 (4)	3 (6)	3 (10)
	Lack of autonomy	A couple of weeks into lockdown I had a meltdown and burst into tears. I just felt overwhelmed with all the sudden change, mixed with pregnancy hormones, and the loss of control over my situation. (A127)	5 (8)	< 1 (1)	2 (9)
	Unmet mothering tasks	When we went into lockdown for COVID-19 I felt a sense of loss and grief for all the things I was no longer able to do with my baby, going to get him weighed, taking him swimming etc. (P107)	1 (2)	1 (2)	1 (4)
**Work and finances**			**12 (19)**	**4 (8)**	**7 (27)**
	Work stress	I’m currently off work due to stress causing depression and anxiety. Everyday life is easier to cope with, but anything involving contact with work makes me extremely anxious. I took ages to fill in some ACAS paperwork about maternity leave and sent it to my line manager. And then I didn’t check my email for a week (because I find it too stressful to do so). When I did I had two replies. One giving me links to my employer’s forms and procedures and another asking if she can pass my personal email address on to one of the HR managers who needs to speak to me. I really couldn’t cope with all the admin (they know what leave I want to take and when from what I sent them) nor can I face a discussion of anything with HR. (A128)	9 (14)	4 (8)	6 (22)
	Financial worries	Household item broke. Got very upset trying to fix it. Started to worry that husband being furloughed and not being able to afford new one I would have to return to work early from my maternity leave. (P142)	3 (5)	0	1 (5)

#### Family wellbeing

Perhaps unsurprisingly, given the context of this research study, the most frequently cited cause of distress was the impact of the COVID-19 virus on their family’s wellbeing. Specifically, participants were concerned with how to avoid the virus:

*‘Needed to go to the hospital for my 20 week scan during the first month of the quarantine/ lockdown. Terrified of going to an area where the virus was mainly and trying to protect my baby in my tummy as well as my 3 year old and husband’* (A142)

This extract typified many of the other comments in the data. Women often positioned themselves as “protectors” with the burden of responsibility regarding their family’s wellbeing falling predominantly on their shoulders. But in the context of the pandemic, where so much is unknown and uncontrollable (particularly in situations where exposure to others was necessary and/or inevitable), the idea of not being able to fulfil this role appeared to generate significant distress and was often described as “terrifying”.

While most responses in the theme were expressed in terms of immediate concerns about contracting the virus, worries about *the long-term implications of social isolation during the pandemic* on their child’s development was also apparent (Table 4).

Outside of the COVID-19 context, more general *fears for their infant’s health, safety and wellbeing* were commonplace and distressing (Table 4). While some fears were grounded in previous personal experiences, others were of hypothetical situations and/or suggestive of a lack of confidence in their own parenting as this respondent described:

*‘When my child was beginning the weaning process and I was so worried about what she was eating and how much she should eat and about choking on food. In the end I just had to tell myself that it was normal to worry and that I have to trust my child to eat what they want’* (P7)

*Obstetric concerns* also presented a significant source of distress to many pregnant people and remained salient to a small number of the postnatal subpopulation (Table 4). These included an array of problems faced during their pregnancy or the immediate postpartum period, such as bleeding in pregnancy, pre-eclampsia, and gestational diabetes. Additionally, many women in the prenatal period were distressed by specific *fears for the pregnancy and birth,* which were frequently associated with a fear of miscarriage. This was often linked to previous traumatic obstetric experiences and/or pregnancy loss, which was described by more than half of the participants contributing to these two sub-themes. For example, this mother explains how previous perinatal bereavement and significant obstetric complications led her to fear for the safety and wellbeing of her current pregnancy:

*‘This is my 3rd pregnancy, my first child died due to placental abruption during labour. My second daughter was born by emergency Caesarean section due to uterine rupture. All my pregnancies are IVF, and this pregnancy was my 3rd and final try. I'm petrified of this baby dying.’* (A134)

It was apparent that for these women, historic trauma was having a considerable impact on their current pregnancy and birth, and a fear of history repeating itself was commonly described, particularly for women who had experienced previous miscarriages.

#### Lack of support

Lack of perceived support during the perinatal period was frequently cited as a source of distress. Some of this was a direct result of COVID-19 restrictions (e.g., lockdown, reduced access to support, changes to perinatal care) which disproportionately impacted the prenatal sub-sample, largely due to the impact of restrictions on prenatal support. The first sub-theme (*isolation and lack of social support*) is characterised by women citing separation from their friends or family as a source of distress. For some, isolation amplified many typical challenges of motherhood, whilst others reported the isolation itself to be their most salient source of distress:

*‘New baby not able to have cuddles from grandparents and support for myself. I got upset when he was uncontrollably crying. It can get too much when there's just me and my husband. If I had my mum who could just come and simply rock my little boy or reassure me that would help massively.’* (P133)

As illustrated by this new mother, her perceived inability to comfort her baby who was ‘uncontrollably crying’ is associated with physical disconnection from her extended family. This participant flags the role of her own mother as an important resource which could have, in pre-pandemic circumstances, mitigated that distress by providing emotional and practical support as she develops confidence as a new mother. The disconnection of the extended family in physical familial spaces is described here as impacting the wellbeing of this new family. This is underscored by the absence of physical demonstrations of connection between family members in which the *“new baby [was] not able to have cuddles from grandparents”.*

The presence of *unsupportive relationships* (the second sub-theme) was also a frequent source of distress (Table 4). Many women felt under-appreciated and undervalued by their partner, becoming frustrated by their partner’s failure to acknowledge the challenges of motherhood. This led to tension and arguments and became more problematic when also struggling with mothering challenges such as sleep deprivation. For some, relationship difficulties extended to significant conflict – *“My husband and I were arguing and I wanted him the leave the house, he wouldn’t and I felt almost panic attack fear that I just didn’t want him near my baby”* (P140). Although domestic violence was not disclosed (despite the increased rates of domestic violence reported during COVID-19 [49, 50]), the psychological impact of dysfunctional relationships was clear, as this account demonstrates:

*‘This morning my partner shouted at me that I was a cunt and told me all I do is whinge and that he doesn't care about me. I cried and thought about hurting myself then told him I want to end the relationship’* (A105)

Some people expressed distress resulting from unsupportive relationships with other family members and friends, however this was less common, and the associated emotions were typically less concerning.

*‘Wanting to avoid groups or circles of people who are less supportive. Specifically, my NCT group from my first baby - I've ignored all the group chats and actually removed most of them on social media as it's impacted my anxiety levels too much.’* (P90)

The third sub-theme, *restrictions in perinatal care,* extended the theme to focus on how restrictions in perinatal care gave way to a lack of support, with pregnant women repeatedly expressing distress resulting from the ban on partners attending routine antenatal appointments, particularly scans. This represented a significant reduction in their perceived support, and many women worried how they would cope if they received bad news at scans alone.

*‘Woke up in the night, couldn’t get back to sleep worrying about whether the baby was ok and what I would do if they told me that the baby wasn’t ok at the scan but my husband wasn’t there’* (A71).

In this extract, hospital restrictions became associated with the mother having to take individual responsibility for handling medical updates. The implicit reference to the support of her husband suggests that the co-parent can mediate this sense of overwhelming responsibility, which when removed, produced pronounced levels of worry.

Others voiced concern over the impact that excluding partners from the antenatal processes may have on paternal bonding with the unborn baby, for example:

*‘I feel alone in the pregnancy as I am unable to take my husband to any scans, I feel worried he won’t bond with his child as he can’t be a part of pregnancy.’* (A122)

As women are socially expected to maintain and facilitate relationship maintenance within families [51], the exclusion of co-partners represents an area that they are socially tasked with but unable to realise in pandemic circumstances.

Several postnatal respondents also reported distress resulting from pandemic-related changes to their care when admitted to hospital:

*‘I was in hospital with my 15 day old baby just a few days again. Because of lock down only I could go in with her, no visitors, no partners, no children, no leaving your room never mind the ward. It was scary, lonely, hard work. ( … ) I had to call in the nurses after 10 mins of crying about the fact I missed my family and couldn't do anything to help.’* (P205)

In this extract, there is a real sense of frustration at not being able to *“do anything”.* This lack of control seems amplified by the lack of access to support from friends and family, and more general freedoms. With visitors prohibited, and mothers confined to their hospital room, women repeatedly reported feeling isolated and alone. They were forced to recover from the physiological experience of birth and care for their new baby without support from coparents, family, and friends - who prior to the pandemic would have been able to visit and provide support.

#### Mothering challenges

Participants often alluded to social expectations of ‘good’ mothering in their responses, which is located within ideals of intensive mothering and remain dominant in Eurocentric societies [52]. Specifically, these ideals position productivity as central to successful parenting in which mothers are expected to devote an inordinate amount of time and energy to child-centred practices to ensure their children thrive [53]. The pressures around managing unrealistic expectations of intensive mothering with mothers’ own needs is well-documented [54].

Efforts to achieve a standard of ‘*good’* mothering and the challenges associated with mothering experiences were frequently cited as a source of distress amongst the postnatal sub-sample. The nature of these challenges varied but fell broadly into three subthemes of ‘*infant crying and sleep deprivation*’, ‘*difficulty achieving personal mothering expectations*’, and *‘competing demands on time’*. People described struggles coping with the demands of an unsettled child (Table 4), and frequently described distressing guilt in their failure to manage their own frustration:

‘*I got really frustrated and shouted at her and then started crying because I felt I was a horrible mother not able to even give my child a routine. Then I felt horrible because I snapped at her.’* (P82)

In the context of unrealistic intensive mothering ideals, ‘good’ mothering is often associated with heavily sanitised versions of the ‘happy’ family in which high quality positive affective engagements are seen to enhance the wellbeing of its members [54]. Here, this mother labels herself as a ‘horrible’ mother, because negativity affectivity is positioned as out of kilter with ‘good’ mothering ideals. There is a clear sense of failure linked to social expectations of what constitutes basic parenting (e.g., “not able to *even* give my child a routine”) and associated feelings of negative affect.

*Competing demands on time* was also a significant source of pressure and distress. With schools and childcare settings closed as a result of the pandemic, and homeworking forced upon many, women frequently struggled to juggle home and work life and felt overwhelmed by the competing demands on their time. Postnatal women described difficulties in caring for a new-born whilst home-schooling older children. Although some benefitted from their partner working from home, others found this added to their difficulties:

‘*Baby failing to settle, other child crying, husband complaining as he was trying to work from home.’* (P118)

The gendered division of labour is made visible in this example, with this mother positioned as responsible for managing the competing needs of children and her partner. Childcare is still commonly seen as predominately women’s work [55] which is reflected in a burgeoning body of literature which suggests that the impact of the Covid-19 lockdowns and school closures was felt more intensely by mothers than fathers [56]*.* As Auðardóttir and Rúdólfsdóttir ([57]) argue, mothering during COVID-19 has been an *“overwhelming project that requires detailed organisation and management”* (p. 1) adding to the existing pressures and anxieties associated with motherhood.

#### Loss of control due to COVID-19

Many respondents reported struggling with a loss of control due to the imposed restrictions on their day-to-day movements, as well as future plans during the perinatal period. Many of the quotes in this theme had an overriding sense of *feeling trapped* by the restrictions that were imposed as a result of the COVID-19 pandemic.

*‘I feel trapped in the house and I feel trapped by my new baby. ( … ) If there was stuff to do or to look forward to but being stuck inside makes it feel like there's no endpoint.’* (P116)

Women often positioned themselves as helpless in the situation, having to obey externally imposed Government restrictions, representing a feeling of powerlessness with women not knowing what to do. This *lack of autonomy* was often evident from the responses, with some women also highlighting their frustration at having to be being dependent on others:

*‘Couldn't get a food shop delivery and this panics me as I can't go to the shops myself and my husband finds it very stressful during the pandemic and I feel useless as I can't do anything.’* (A75)

In this extract, the participant describes a common experience in the height of the pandemic concerned with the rapid booking and unavailability of online food shopping delivery slots. Her feelings of panic are described as arising from a loss of independence from being able to perform a basic and routine household activity (food shopping) and the impact of her newfound dependency on her husband who “finds it very stressful”. Dependency here is constituted as burden for her husband which reinforces her sense of uselessness.

Resonant with the above extract, many of the responses also referred to the loss of normality arising from the lockdowns as a source of their distress. This included the loss of being able to do typical and taken-for-granted tasks. This can be seen in the following extracts:

*‘Not being able to go to the supermarket as I would have before Covid-19’* (A163)

*‘feeling of isolation and not being able to continue usual routine’* (A129)

The loss of the normal expected maternity experiences was also identified as a cause of new mothers’ distress.

*‘I felt helpless, like I didn’t know what to do with myself, my time, my baby. I felt I had lost myself and had nowhere to go and nothing to aim for or make plans for. I cried uncontrollably and had no energy to do anything but feed and hold my baby. I was stagnant - this was not what I envisioned new motherhood to be like.’* (P73)

In this quote there is a sense that the impact of restrictions on this new mother are profound. Not only does she feel physically restricted (having *nowhere to go*), but the restrictions are also described as creating a new and deeply negative reality for new motherhood that stood as a stark contrast to imagined and expected maternity experiences in pre-COVID-19 contexts. This mismatch between perinatal reality and expectations have previously been shown to be a significant source of perinatal distress [11], which is also highlighted here. In this case, the participant describes herself as helpless to change the situation, causing significant distress. Indeed, the COVID-19-related restrictions were described as causing particular distress for mothers in terms of being able to do the things they wanted to for their children:

*‘it is my youngest son's birthday on Saturday and I felt very upset that he won't be celebrating it like he would normally, there is no party or anything fun, and I feel like I'm letting him down because of it.’* (A186)

In line with intensive mothering ideals mentioned earlier, women are socially expected to invest an inordinate amount of resource and labour (time, emotional, financial) in their child-centred parenting to ensure that their children thrive. The lockdowns curtailed the ability to intensively mother in ways expected pre-COVID-19. Not being able to fulfil this role by carrying out these *mothering tasks* due to restrictions imposed caused them significant upset and guilt, despite having no control over the situation (and the situation not being their fault). Descriptions of loss and guilt here illustrate how deeply entrenched such mothering ideals and expectations are and how they shape parental experiences of distress.

#### Work and finances

A final theme highlighted sources of distress related to work and finances, predominantly experienced by the prenatal sub-sample (see Table 4). It is unsurprising that this source of distress was more frequently reported by pregnant people, given that many postnatal participants were not working at the time of responding to the survey (Table 1). As with other themes, the context of COVID-19 exacerbated work and financial stress.

Whilst some women expressed concern regarding employment associated with COVID-19 working restrictions (Table 4), others described the practical difficulties of adapting to working through a pandemic:

*‘This morning when working online at home (as a teacher) and a parent was moaning. I felt like my head of pre-prep had let me down. I phoned her to discuss what she has told parents was expected of me, I felt it was too much. In the end I had to leave a voicemail and cried at the end (I tried to hold it together). As I am pregnant I cannot teach my year one class in school, but feel like I am being punished for this as the expectations to teach live to my class in school, and then those at home, I feel this is unmanageable.’* (A3)

In this extract, the participant describes becoming distressed as a result of expectations around what she *should* do as a professional (teach live) directly conflicting with what she is *able* to do (I cannot teach … in school). The described unreasonableness of others’ responses (parental compliant, lack of support from superordinate) to her choice of managing her pregnancy safely in the pandemic context is constituted as overwhelming and “unmanageable” particularly when contextualised within the wider pressures, “those at home” impacting the participant.

Many postnatal women seemed torn between their professional identity and their relatively new identity as a mother. In some cases, it was evident that the mothers’ work identity and their relationship with their colleagues was important to them, but they needed to balance this with a desire to protect their baby.

*‘I felt like I should be back helping my colleagues but worried I would bring the virus home to my baby.’* (P154)

In other cases, mothers did not feel ready to be separated from their baby and return to work:

*‘I had a telephone call scheduled with my manager (my maternity leave is about to end) which made me very panicky and stressed - not the call itself but more the thought of being closer to going back to work and being apart from my baby.’* (P58)

Financial issues also caused distress in this study. Sometimes this was inextricably tied up with work concerns, and a direct result of COVID-19 and the associated job losses and/or furlough scheme.

*‘Household item broke. Got very upset trying to fix it. Started to worry that husband being furloughed and not being able to afford new one I would have to return to work early from my maternity leave.’* (P142)

In the above extract, the participant’s upset is constituted as grounded in the worry that she would be forced to prematurely end her maternity leave, and her time devoted to mothering her child, because of new financial pressures. In other cases, this was due to mothers feeling that they may not be able to provide everything they would like for their baby:

*‘I was shopping for things for the baby when I realised we couldn't afford to buy all the things that I would like because of the current situation.’* (A50)

Here, the participant describes a sudden realisation of her family’s financial situation that is brought on during the act of shopping. Her current situation, beyond her control, places limits on her ability to both exercise choice over what she buys and to provide for her baby.

## Discussion

Our study qualitatively explored the feelings and symptoms perinatal women associated with psychological distress during the COVID-19 pandemic, and shed light on the sources of these experiences. We now consider our synthesised findings in relation to previous research and highlight possible opportunities to support perinatal women within and beyond the COVID-19 pandemic.

### Feelings and symptoms associated with perinatal distress

In keeping with previous research [12], tearfulness was the most frequently expressed feeling or symptom associated with distress, reported by almost half of the participants. Anxiety-related symptoms of ‘worry and overthinking’, ‘fear’, ‘anxiety and nervousness’, and ‘panic’, were, when combined, also described by more than half of the respondents. This was not surprising given PASS scores suggested a similar proportion of the sample scored over the cut-off for clinically significant anxiety symptoms. ‘Worry and overthinking’ was the second most reported symptom category associated with distress, in contrast with previous work that found ‘worry and fear’ to be rarely described as a symptom of perinatal distress [12]. This difference may reflect the context of the pandemic, as several factors specifically related to COVID-19 may have increased the salience of worry in the present sample (e.g., [25, 58]). Alternatively, this discrepancy could be the result of methodological differences. For example, Coates et al. [12] analysed a small number of in-depth interviews, providing opportunity for an array of symptoms to be expressed; in comparison, survey questions (as used in the present study) typically elicit a brief response. Furthermore, differences may be due to the way symptoms were clustered (Coates et al. treated ‘overthinking’ as an independent theme and clustered ‘worried’ with ‘scared’, while we combined ‘worry and overthinking’) and participant profiles (fear was more commonly reported amongst prenatal women than postnatal women in our analysis, however this group was not included in Coates et al.’s research).

Whilst our prenatal and postnatal sub-samples contained a similar proportion of participants scoring above the threshold for perinatal anxiety and depression, the feelings and symptoms they described in relation to their distress differed. For example, guilt, failure, self-blame, and inadequacy were more commonly reported by postnatal women. In contrast, our prenatal subsample more often reported fear, which was typically associated with pregnancy-specific experiences, such as the fear of miscarriage. This accords with research that suggests pregnancy anxiety should be recognised as a unique construct [59, 60]. Thus, more research is needed to explore whether screening tools and interventions designed to identify and support perinatal people may benefit from targeting the feelings and symptoms most relevant to each period, rather than treating them as one homogeneous group.

Despite the known comorbidity of perinatal anxious and depressive symptoms [8], and the prevalence of clinically relevant depressive symptoms in the present sample (Table 2), the feelings and symptoms linked to distress were more typically associated with anxiety, as well as transdiagnostic signs and symptoms of psychological distress, rather than those traditionally related to depression. This is an important finding considering that most PMH information and support is focused on symptoms and feelings associated with postnatal depression [11, 12], and a lack of understanding of the common signs and symptoms of other PMH difficulties is a known barrier to support [15]. Thus, PMH information should capture the true array of feelings and experiences associated with psychological distress if people are to identify their difficulties and access the support they need [11]. For example, given the high levels of perinatal anxiety documented in the literature [61] and seen in this study, future research, screening, and support may benefit from further focusing on this construct. Additionally, it has been proposed that focusing on broader expressions of perinatal distress (such as pregnancy- specific stress [62]; a transdiagnostic construct linked to maternal mental health and birth outcomes) may be more beneficial than focusing on the specific disorders of perinatal anxiety and depression [12, 16].

### Sources of perinatal distress

Five themes (*Family wellbeing; Lack of social support; Mothering challenges; Loss of control due to COVID-19; Work and finances*) and seventeen sub-themes (see Table 4) captured the array of events and experiences that perinatal women associated with their feelings of distress. The impact of the pandemic was evident across the themes and broadly echoed findings from elsewhere in the world during the pandemic (e.g., [28–31]). For example, fears about contracting the virus, and restrictions on social interactions, perinatal care and movements were clear sources of distress, as was the sense of loss of control the situation gave rise to. However, many of the themes and sub-themes related to more general aspects of perinatal experience.

Fear for the wellbeing of family members, in relation to the potential short and long-term consequences of COVID-19, and a general fear for infant wellbeing (unrelated to COVID-19), were common sources of distress. When pregnancy fears were reported, they were often associated with what appeared to be unresolved trauma of historic obstetric events, such as miscarriage and pregnancy complications. This may reflect signs of Childbirth-Related Post-Traumatic Stress Disorder (CR-PTSD), and/or secondary Tokophobia which previous research has identified as a concept uniquely experienced by women [63, 64]. Our findings reiterate the need for further research into these constructs to better understand their prevalence, identification and associated risk factors, and inform the development of interventions to support individuals most at risk [64]. The present findings also contribute to a large body of evidence pertaining to pregnancy-specific anxiety (e.g., [59, 60, 65]), and suggest improved availability of support and information around specific fears for the pregnancy and infant wellbeing may alleviate some distress not necessarily related to previous experiences. Themes also revealed the importance of co-parents in supporting women through pregnancy, particularly when specific pregnancy fears were described, reinforcing the need to include co-parents in perinatal primary care.

This research enriches understanding of the role social relationships play in supporting PMH (e.g., [5, 6, 35, 36, 66–68]). At the time of data collection, lockdown restrictions in place to mitigate the spread of COVID-19 forced families into physical social isolation. Being unable to spend time with friends and extended family was commonly attributed to feelings of psychological distress, particularly within the postnatal subsample. Mothers also expressed concern regarding lost opportunities for their infant to build relationships with their wider family. This echoed concerns surrounding the loss of social support, bonding rituals, and traditional birth celebrations reported in research conducted in Australia [28] and the USA [29].

It is important to note that not all participants described beneficial social relationships, as some described the role unsupportive relationships can play in psychological distress. One in ten respondents attributed their distress to dysfunctional interactions with their partner, family, or friends. Indeed, extracts presented earlier highlight concerning examples of significant distress resulting from relationship conflicts. This accords with evidence of relationship dissatisfaction being a significant risk factor for perinatal distress [69–72]. Furthermore, although not disclosed in the present dataset, domestic violence is reported to have increased during the COVID-19 pandemic [49, 50] and should always be considered in practice when people disclose distress associated with close relationships.

Across the themes, but most visible in the ‘Mothering challenges’ theme, mothers positioned themselves as providers and/or facilitators for their children, reflecting the gendered division of responsibility and labour across familial roles. This was also evident in the apparent conflict women described between their professional and maternal identities, where the latter often took precedence over the former. This positioning often functioned as a source of distress, when women felt they were unable to fulfil their mothering role. Additionally, the experienced mismatch between expectations of pregnancy or motherhood and reality, and an associated guilt for not achieving contemporary (unrealistic) ideals around ‘*good’* mothering, was repeatedly observed, both in relation to pandemic-specific stressors, and general perinatal experiences. These findings are consistent with the results of previous qualitative research which have attributed unrealistic expectations of motherhood, and guilt and self-blame, to psychological difficulties in the postnatal period [11, 16, 73]. This also fits with quantitative evidence of maladaptive beliefs towards motherhood increasing the risk of perinatal anxiety and depressive symptoms [74–76], and the reported relationship between dysfunctional perfectionism and postnatal distress [77]. As such, managing misconceptions around mothering ideals and better preparing people for the challenges of pregnancy and motherhood, such as infant crying and sleep deprivation, may be helpful in reducing perinatal distress.

A sense of lack of control was also evident across the themes, whether it was feeling like they were powerless to meet their child’s needs in some way (e.g. being unable to comfort “uncontrollable” crying, alleviate distress or ill health, or financially provide for them in the way they wanted), or conveying a sense of helplessness in the face of the pandemic and the restrictions that were imposed as a result of it (i.e. limitations on social interactions and movement). Regardless, this lack of agency was often implicitly or explicitly described as giving rise to significant frustration and distress, consistent with previous work that has highlighted lack of control as a contributing factor to perinatal mental health issues [11].

### Strengths and limitations

When interpreting these findings, it is important to consider several limitations. Firstly, the self-selected convenience sample lacked diversity (most participants were white, highly educated, women in normative relationships), and as such may not be representative of the UK population. In addition, participants’ responses may have been influenced by social-desirability biases. Conversely, biases towards disclosure surrounding mental health issues may have existed in the sample, as women particularly affected by these issues may have been more likely to take part. Thus, it is unclear how generalisable, or representative these findings may be. However, Braun et al. [40] point to the benefits of anonymous questionnaires when researching sensitive subjects, and Moore et al. [78] proposed that online data collection methods may encourage disclosure of PMH difficulties. Second, the single open-ended survey question provided limited access to participants’ experiences, although it allowed for the inclusion of a larger sample than many other qualitative methods, which was a significant strength. Relatedly, we relied exclusively on self-report measures; future studies could usefully include detailed interviews to yield a richer understanding of perinatal women’s experiences of distress. Third, the framing of the question may not have prompted information desired to answer the specific research questions, particularly with regards to feelings associated with distress where feeling upset may have been expressed to mirror the survey question which could explain its frequency. However, the indirect nature of the question prevented wording bias and allowed women to share the thoughts and experiences most salient to them. Fourth, researcher bias is always possible in qualitative studies, however prior awareness of these effects meant effort was made to contain biases. Finally, findings are embedded within the context of the COVID-19 pandemic, nevertheless many of the concerns and experiences reported by participants were not related to COVID-19, and may be useful to generate hypotheses for future research and expand the knowledge base relating to perinatal distress, potentially enabling healthcare professionals to better respond to patient needs.

## Conclusions

This study identified feelings and symptoms reported to accompany perinatal distress, highlighting a range of emotions associated with distressing experiences. Although findings are couched in the context of COVID-19, they have the potential to guide further investigations and provide important insight into the experiencse of pregnant and postpartum women. Nonetheless, further research is recommended to confirm that these findings are relevant beyond the pandemic. Our exploration of the psychosocial sources of perinatal distress revealed key areas in which perinatal people could be better supported. For example, holding unrealistic expectations about perinatal experiences and what constitutes ‘good’ mothering identities appear to be problematic when these ideals cannot be met. As such, promoting more realistic expectations of motherhood, and altering the standard women hold themselves to may reduce feelings of guilt and failure which can be associated with perinatal psychological distress. The results also highlight the importance of maintaining key sources of social support throughout the perinatal period, and in particular, suggest that co-parents should be included in perinatal care wherever possible. Our findings also suggest specific attention should be paid to pregnancy-related fears, particularly in people who have previously experienced traumatic obstetric events. When a birthing person perceives an obstetric event to be traumatic, psychological support may be beneficial, and may have the potential to reduce the risk of it affecting emotional wellbeing in the event of future pregnancies. Moreover, several sub-themes specifically related to the context of COVID-19 point to opportunities to reduce distress should further social restrictions be necessary. Over the coming years it will be important to remain aware of the distress experienced during the pandemic and reflect on how this may project onto future perinatal experiences. Indeed, the perinatal cohort of 2020/1 may benefit from additional support to prevent enduring psychological distress, particularly given the high rates of probable depression and anxiety in the current sample.

## Data Availability

Anonymised versions of the datasets used and analysed during the current study are available from the corresponding author on reasonable request.

## Abbreviations

CR-PTSD: Childbirth-Related Post-Traumatic Stress Disorder
EPDS: Edinburgh Postnatal Depression Scale
PASS: Perinatal Anxiety Screening Scale
PMH: Perinatal Mental Health
